# Climatic Control on Plant and Soil δ^13^C along an Altitudinal Transect of Lushan Mountain in Subtropical China: Characteristics and Interpretation of Soil Carbon Dynamics

**DOI:** 10.1371/journal.pone.0086440

**Published:** 2014-01-23

**Authors:** Baoming Du, Chunjiang Liu, Hongzhang Kang, Penghua Zhu, Shan Yin, Guangrong Shen, Jingli Hou, Hannu Ilvesniemi

**Affiliations:** 1 School of Agriculture and Biology and Research Center for Low-Carbon Agriculture, Shanghai Jiao Tong University, Shanghai, China; 2 Key Laboratory of Urban Agriculture (South), Ministry of Agriculture, People’s Republic of China, Shanghai, China; 3 Instrumental Analysis Center of SJTU, Shanghai Jiao Tong University, Shanghai, China; 4 Finnish Forest Research Institute, Vantaa, Finland; North Carolina State University, United States of America

## Abstract

Decreasing temperature and increasing precipitation along altitude gradients are typical mountain climate in subtropical China. In such a climate regime, identifying the patterns of the C stable isotope composition (δ^13^C) in plants and soils and their relations to the context of climate change is essential. In this study, the patterns of δ^13^C variation were investigated for tree leaves, litters, and soils in the natural secondary forests at four altitudes (219, 405, 780, and 1268 m a.s.l.) in Lushan Mountain, central subtropical China. For the dominant trees, both leaf and leaf-litter δ^13^C decreased as altitude increased from low to high altitude, whereas surface soil δ^13^C increased. The lower leaf δ^13^C at high altitudes was associated with the high moisture-related discrimination, while the high soil δ^13^C is attributed to the low temperature-induced decay. At each altitude, soil δ^13^C became enriched with soil depth. Soil δ^13^C increased with soil C concentrations and altitude, but decreased with soil depth. A negative relationship was also found between O-alkyl C and δ^13^C in litter and soil, whereas a positive relationship was observed between aromatic C and δ^13^C. Lower temperature and higher moisture at high altitudes are the predominant control factors of δ^13^C variation in plants and soils. These results help understand C dynamics in the context of global warming.

## Introduction

In terrestrial plant ecosystems, ^13^C enrichment is generally greater in the soils than in vegetation due to the different fractionation of ^13^C and ^12^C in the biogeochemical processes [1], [2]. In the last three decades, the C isotope technique has been a useful approach for understanding the effects of water deficit on plants [3], [4], dynamics of soil organic C (SOC) [5], [6], [7], and local climate history [8]. δ^13^C has been used as an index to study ecosystem response to climate and as a surrogate variable in modeling C fluxes in terrestrial ecosystems [9], [10].

At a site scale, soil δ^13^C is closely associated with local vegetation and climate history, and varies with the soil depth owing to its longer-term fractionation in deeper soil [11]. The impact of vegetation on soil δ^13^C could vary with plant species and tissues. For instance, C_3_ (Calvin cycle photosynthetic pathway) plants have δ^13^C values of –22‰ to –35‰ (average –26.5‰), compared to C_4_ plants whose δ^13^C values are in the range of –8‰ to –16‰ (average –12.5‰) [8], [12]. This difference help determine the relative proportion each vegetation type in soil organic C [13], [14]. Among plants tissues, δ^13^C is typically enriched by 1‰ to 2‰ in celluose and hemicelluloses, but depleted in ^13^C by 2‰ to 6‰ in lignin, in comparison with whole-plant material [6], [15].

At a broader scale, soil ^13^C varies with spatial gradients by altitude, latitude, longitude due to changes of vegetation and climate [16]. Bird et al. (1996) reported that the average δ^13^C was –28.3±0.6‰ in low latitude (0°N to 20°N or S), –27.7±0.6‰ in mid latitude soils (20° to 40°), and –27.3±0.7‰ in high latitude soils (40° to 90°) [17]. The overall increase of SOC δ^13^C from low to high-latitude forests was −1‰. In mountain areas, climate varies with altitude resulting in different vegetation, litter input into soils, and litter decomposition rate, and therefore soil δ^13^C. Wei and Jia (2009) reported that soil δ^13^C first decreased and then increased as altitude increased from 1000 m to 3800 m in Mount Gongga, southwestern China [18]. Consequently, ecosystems in mountain areas are very sensitive to changes in climate [19], [20].

Lushan Mountain is located in the middle-lower plain of the Yangtze River in central subtropical China, with altitude from 30 m a.s.l. to 1470 m a.s.l. [21]. Along with the change of elevation is the opposite trend of heat and water (OHW), i.e., temperature decreases and precipitation increases from low to high elevations. Correspondingly, vegetation changes from evergreen broadleaf forests at the foothill to deciduous forests at the top of mountain. The OHW is a common mountain climate in subtropical China and has unique community composition, litterfall decomposition ratio, soil C biochemical processes for exploring the variations of plant and soil δ^13^C with altitude, particularly in relation to climate change.

In this study, we examined the variation of δ^13^C in fresh plant leaves, litter, and semi-decomposed and soil humic substances in order to characterize soil carbon dynamics at a local or regional scale in subtropical forest ecosystems in Lushan Mountain, China. The specific objectives were to: 1) determine the patterns of δ^13^C variation in dominant plant species and soil with altitude, 2) investigate how soil δ^13^C varies with soil depth at different altitudes, and 3) examine the effects of climatic factors on plant and soil δ^13^C along the elevation gradient in Lushan Mountain.

## Materials and Methods

### Study Area

**Figure 1:**
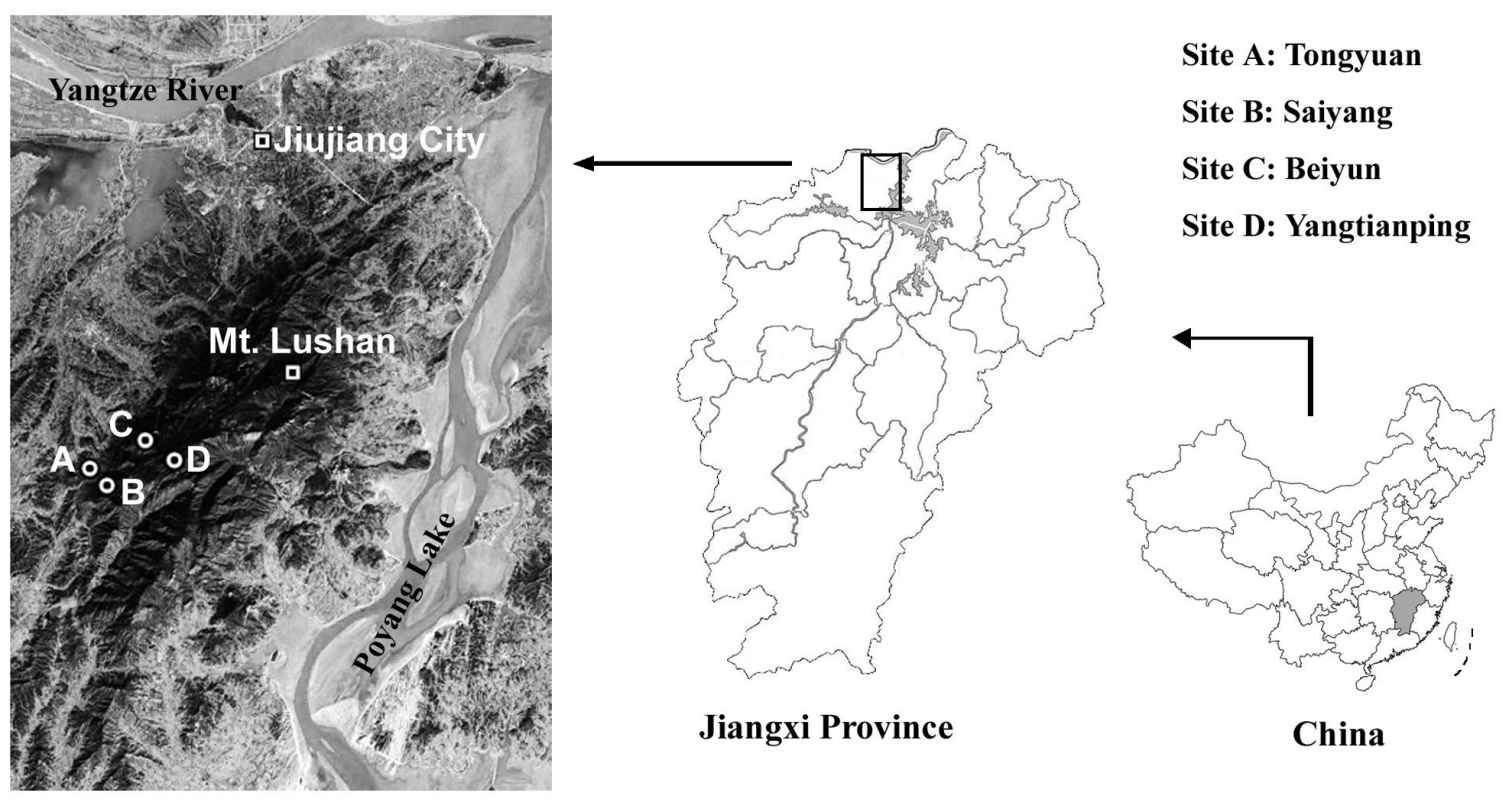
Location of the study area and the distribution of sample stands in Lushan Mountain, subtropical China.

Study area is located in the Lushan Nature Reserve (29°31′–29°41′N, 115°51′– 116°07′E), the south of Jiujiang City, Jiangxi Province, China (Fig. 1). Permission was granted by the Nature Reserve of Lushan Jiangxi. Lushan is an isolated mountain body situated in the center of the vast plain of the middle and lower reaches of the Yangtze River, covering an area of about 300 km^2^ along a altitude range from 50 m to 1474 m. This area has a subtropical monsoon climate. The mean annual precipitation (MAP) ranges from 1308 mm to 2068 mm, and the mean annual temperature (MAT) from 17.1°C to 11.6°C [21].

Owing to the variations in geology, climate, and vegetation along elevation, dominant soil types change from Ferric alisols at low elevations to Haplic alisols at high elevations [21] according to the FAO soil texture classification. The corresponding vegetation types are evergreen forests dominated by several Fagaceae tree species including *Castanopsis sclerophylla*, *Castanopsis eyrei* and *Lithocarpus glaber*, and some evergreen woodland species and shrubs at low altitudes of approximately 50 m to 600 m, evergreen broadleaf forests and some deciduous trees are mid altitudes of 600 m and 1000 m, and *Lindera obtusiloba* forest consisting of *Cerasus serrulata*, *Castanea seguinii*, *Tilia breviradiata*, and a few shrubs at about 1200 m.s.l. (Table S1). Some *Cryptomeria japonica* plantations were established about 50 years ago at mid altitudes.

### Sample Stands and Collection

Permission was granted by the Nature Reserve of Lushan Jiangxi (29°31′–29°41′N, 115°51′–116°07′E) to carry out our study. Sample stands were chosen at an irregular altitude interval owing to natural forest fragmentation in mountain. In total, four study sites were established at altitudes of 219 m, 405 m, 780 m, and 1268 m (Fig. 1, Table 1). These study sites were not associated with endangered or protected species.At each site, three plots (each 20×20 m) were randomly delineated. In each plot, four 4 m^2^ subplots were randomly chosen for shrub layer and four 1 m^2^ subplots for herbaceous layer. In each plot, 100 leaves were collected from dominant tree (Table S1). Sample were collected from L (Litter) and LF (Semi-decomposition litter) horizons and mineral soil layers at 0–10 cm, 10–20 cm, 20–30 cm, 30–40 cm, 40–50 cm, and 50–60 cm depths. Five soil cores were randomly collected within each plot using a 2 cm-diameter stainless steel borer and bulked to make one composite sample by soil depth.

**Table 1:** Features of climate and vegetation at different altitudes in Lushan Mountain.

Location	Altitude (ma.s.l.)	MAP^a^(mm)	MAT(°C)	TCM^b^(°C)	THM^c^(°C)	Growing season (Days)	Vegetation types^d^
Tongyuan	219	1429	16.2	3.8	28.5	262	EBF
Saiyang	405	1549	15.3	3.2	27.4	253	EBF
Beiyun	780	1794	13.6	1.9	25.1	234	EBMF
Yangtianping	1268	2112	11.3	0.3	22.2	209	DBF

a The climatic data from years 1971 to 2000 were obtained from the Lushan Meteorological Bureau.

b Temperature of the coldest month.

c Temperature of the hottest month.

d EBF represents for evergreen broadleaf forest; EBMF for evergreen broadleaf and needle-leaf mixed forest; and DBFfor deciduous broadleaf forest.

Soil samples were air dried, ground, and passed through a 2 mm sieve to remove coarse living roots and gravel before being ground and passed through a 0.149 mm mesh sieve prior to chemical analysis. Leaf and litter samples were oven dried (65°C) for a week to constant weight and ground to fine powder using a Tecator sample mill (Subang, Shanghai, China) prior to the chemical and isotopic analyses.

### Chemical Analysis

The C isotope ratio (δ^13^C) of leaf, litter, and soil samples was determined using an elemental analysis–stable isotope ratio mass spectrometer (VarioElIII/Isoprime, Elementar, Hanau, Germany) operated at the Instrumental Analysis Center of Shanghai Jiao Tong University (SJTU). The results are reported as parts per thousand (‰) deviations from the Vienna–Pee Dee Belemnite (PDB) standard (uncertainty of ±0.1‰ uncertainty), which The is expressed as follows:
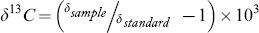



Where δ_sample_ is the^ 13^C/^12^C ratio of the samples and δ_standard_ is the ^13^C/^12^C ratio of the reference standard (PDB) [22].

To express the absolute variation of soil δ^13^C enrichment relative to litter, we define an absolute enrichment factor *F*_A_ as follows:
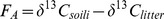
Where δ^13^C_soil *i*_ is the δ^13^C at *i*th soil layer and δ^13^C_litter_is the δ^13^C at the litter layer.

The rate of soil δ^13^C enrichment varies with soil depth [1], [2]. To express the relative enrichment of adjacent soil layers, we define the relative soil δ^13^C enrichment factor *F*_R_ as follows:
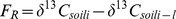
Where δ^13^C_soil *i*_ is the δ^13^C at *i*th soil layer and δ^13^C_soil *i-1*_ is the δ^13^C at *i-1*th soil layer.

To explore the relationship between soil δ^13^C and SOC concentration, as well as soil C functional groups, two soil variables were determined. The variation patterns in SOC concentration and soil C functional groups along the altitude gradient in Lushan Mountain will be presented in another paper. The SOC concentrations of soil samples from different depths were measured using dichromate oxidation method [23].

The chemical compositions of C in litter and soil layers at 0–10 cm, 30–40 cm, and 50–60 cm depths were analyzed with solid-state ^13^C cross-polarization magic angle spinning–nuclear magnetic resonance (CP/MAS–NMR). The litter samples were dried to constant weight at 65°C and ground in a Wiley mill. The soil samples were pretreated with 10% (v/v) hydrofluoric acid (HF) before the NMR spectroscopy [24] to reduce Fe^3+^ and Mn^2+^ [25] and concentrate organic C for more accurate signal-to-noise ratio [24]. About 10 g of the ground sample was shaken with 50 ml HF for 2 h. After centrifugation (5,000 rpm) for 10 min, the supernatant was removed. The procedure was repeated five times. The remaining sediment was washed five times with 50 ml deionized water to remove residual HF before freeze drying.

The solid-state ^13^C CP/MAS–NMR spectra of litter and soil samples were obtained at a frequency of 100.64 MHz using a Bruker AVANCEIII400 NMR spectrometer (BrukerBiospin, Rheinstetten, Germany) operated at 75.42 MHz for ^13^C. The contact time was 1.5 ms, with 1 s recycle delay and the magnetic angle spinning rate was 5 kHz [24]. About 12,000 scans were collected for soil samples and 10,000 scans for litter samples [26]. The chemical shift regions 0–45 ppm, 45–110 ppm, 110–160 ppm, and 160–220 ppm were assigned to alkyl C, O-alkyl C, aromatic C, and carboxylic C, respectively [24], [27]. The sources of organic carbon are: Alkyl C is derived from lipids, fatty acides and plant aliphatic polymers, O-alkyl C primarily from cellulose and hemicelluloses, as well as starch, proteins and carbohydrates, aromatic C from lignin and tannins, and carboxyl C from lipids, aliphatic esters, and amide carboxyls [28], [29]. The signal intensities in the respective chemical-shift regions were expressed as a percentage of the area of the total spectra. The relative contents of different chemical structures were therefore calculated [26].

### Statistical Analysis

Arithmetic means and standard deviation were calculated. A *t* test (i.e., least significant difference) was conducted to compare the means with a probability level of 0.05 for detecting significant differences. Linear regression analyses were used to examine the relationships between soil δ^13^C and MAT, MAP, and SOC concentrations. All analyses were performed through SigmaPlot 10.0 (Systat Software, Richmond, CA, USA) and SAS V8.1 (SAS Institute Inc., Cary, North Carolina).

## Results

### Variations of Leaf, Litter, and Soil δ^13^C with Altitude

**Figure 2:**
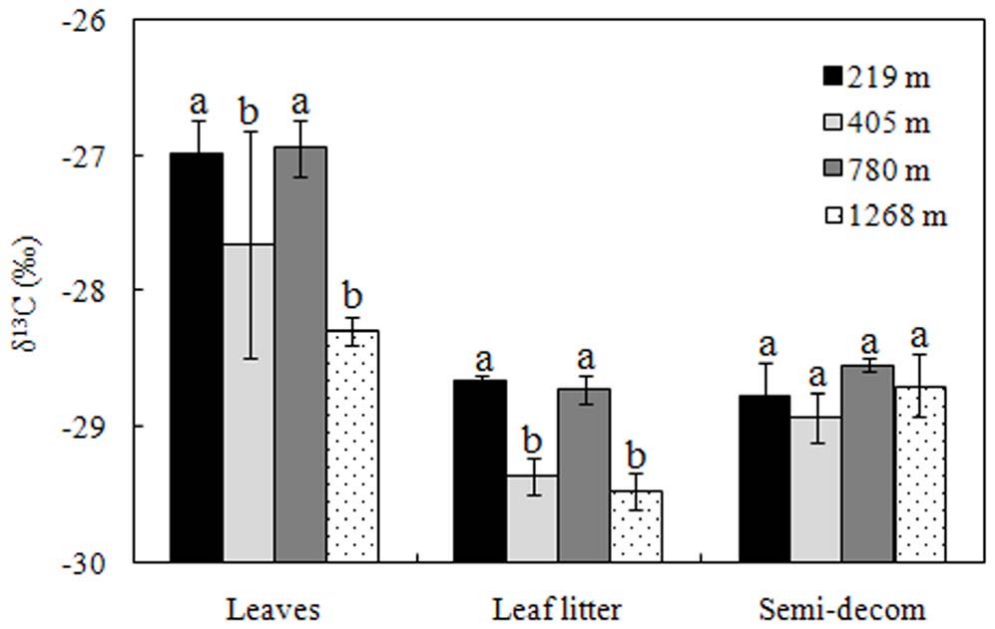
δ^13^C values of leaves, leaf litter, and semi-decomposed litter in the natural secondary broadleaf stands at altitudes of 219, 405, 780, and 1268 m in the Lushan Mountain. The error bars are the standard errors (n = 3 for leaf litter and semi-decomposed litter, and n = 3 for 300 leaves). Different letters indicate significant differences among all classes at the different altitudes (*P*<0.05).

The deciduous tree leaf δ^13^C at 1268 m a.s.l. was –28.29‰, significantly lower than evergreen trees at lower altitudes (–27.65‰ to −26.98‰) (Fig. 2). A similar δ^13^C-altitude relationship was also evident in leaf litter, but not in semi-decomposed litter. Comparatively, fresh leaves had higher δ^13^C than leaf litter or semi-decomposed litter. For instance, δ^13^C decreased from –26.98‰ in fresh leaves to –28.65‰ in leaf litter at 219 m a.s.l. (Fig. 2).

**Figure 3:**
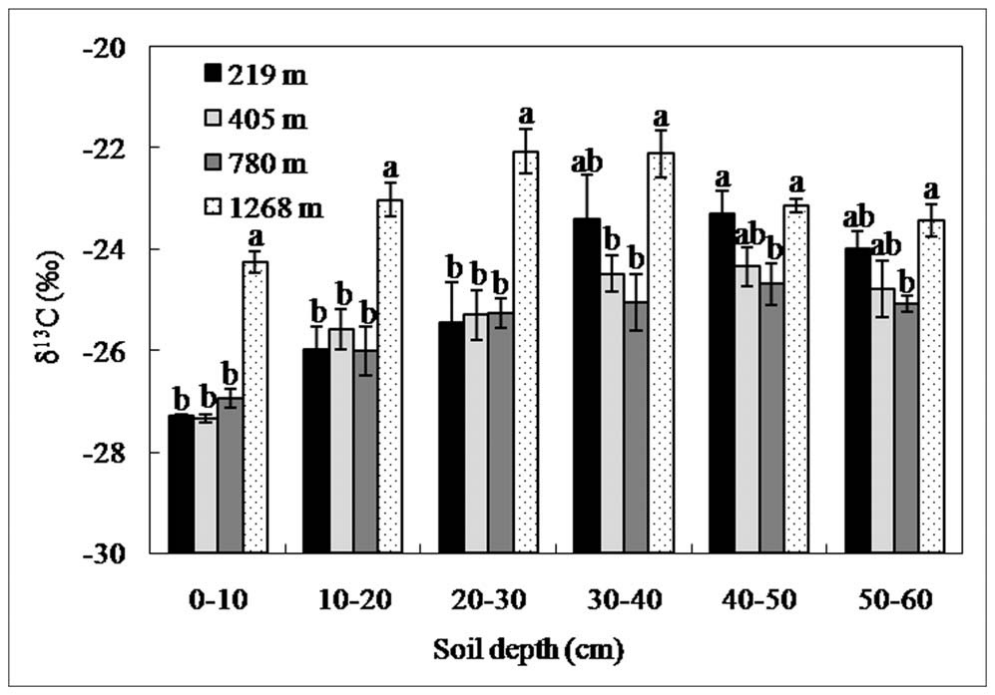
Soil δ^13^C values of different layers in the natural secondary broadleaf stands at altitudes of 219, 405, 780, and 1268 m in the Lushan Mountain. The error bars represent standard errors means (n = 3). Different letters indicate significant differences among altitudes by soil depth (*P*<0.05).

The surface layers of soils (0–10 cm, 10–20 cm, and 20–30 cm) at 1268 m was significantly higher than that at the other three lower altitudes. Ihe deeper soil layers (30–40 cm, 40–50 cm, and 50–60 cm), however, no significant difference by altitude occurred (Fig. 3).

### Soil δ^13^C Enrichment with Soil Depth

**Figure 4:**
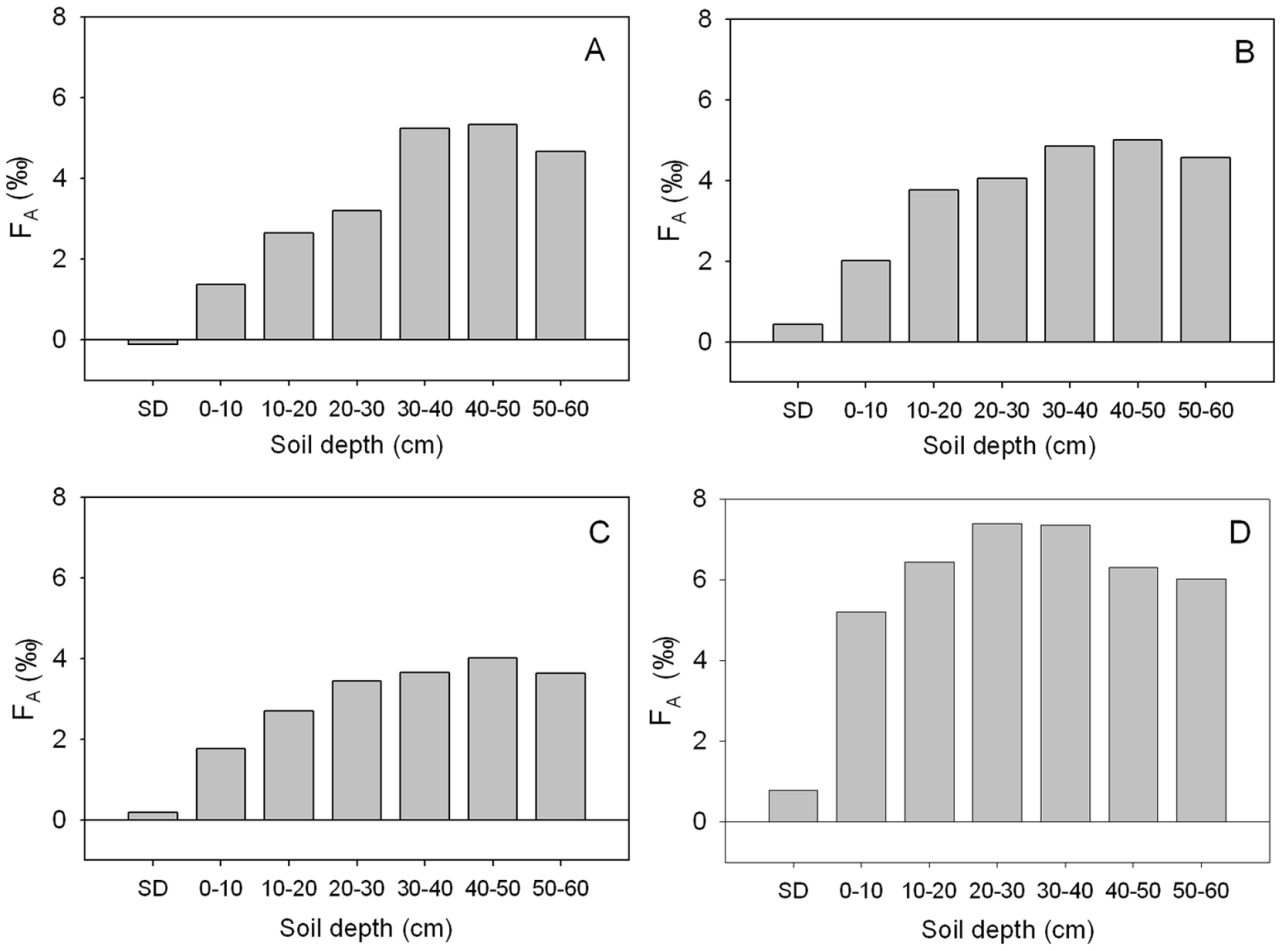
Absolute enrichment factor of soil δ^13^ (*F*_A_) by soil depth at altitudes of 219 (A), 405 (B), 780 (C), and 1268 m (D) in the Lushan Mountain. In the figures, SD represents the semi-decomposed litter layer.

**Figure 5:**
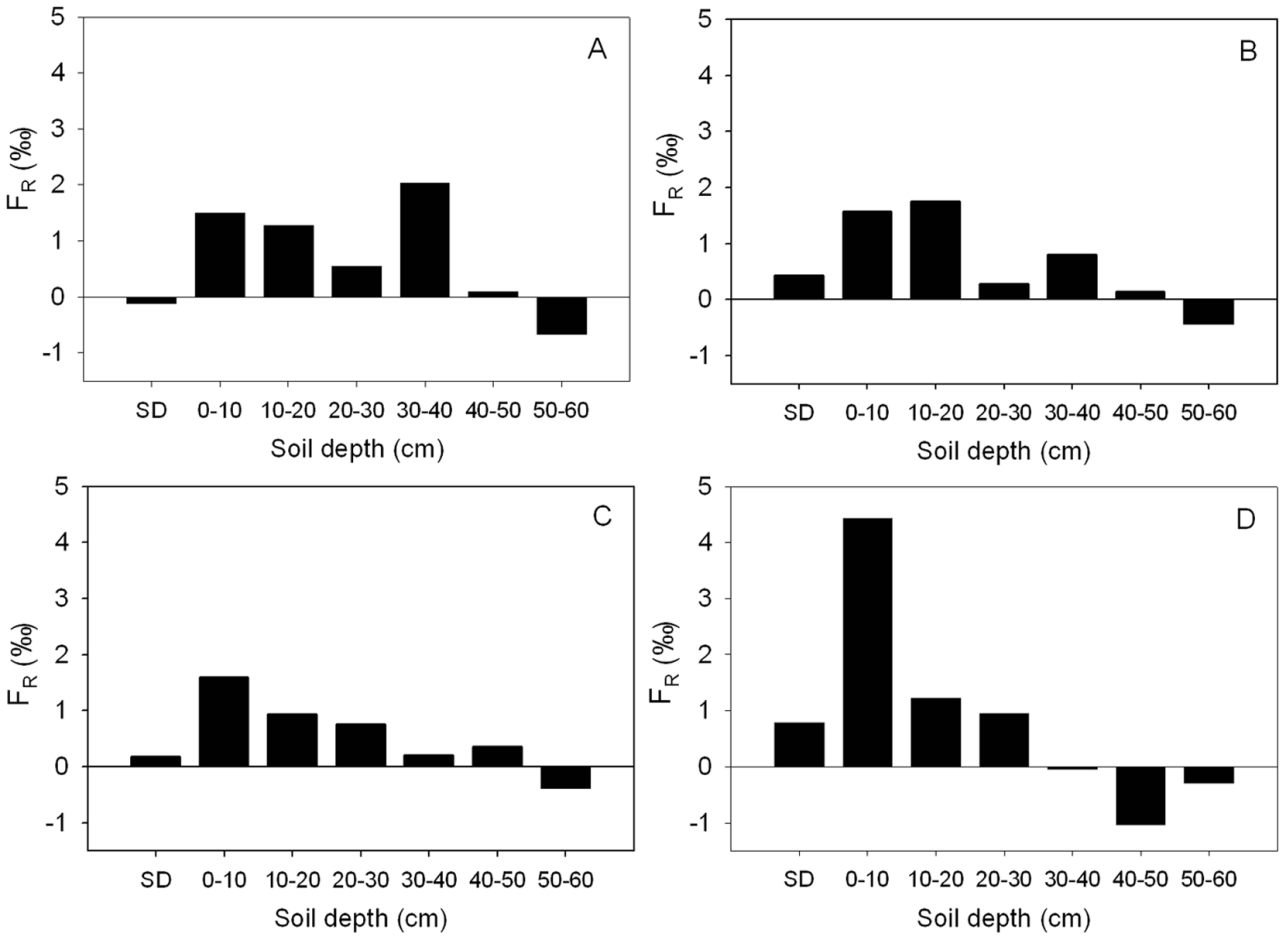
Relative enrichment factor of soil δ^13^ (*F*_R_) by soil depth at altitudes of 219 (A), 405 (B), 780, (C), and 1268 m (D) in the Lushan Mountain.

Soil δ^13^C enrichment was greater with the increase of soil depth for all altitudes (Fig. S1). Absolute enrichment factor (*F*_A_) generally increased from semi- decomposed layer, peaked at 30–40 cm, and remained stable at deeper soil (Fig. 4). The soil *F*_A_ at 1268 m were generally greater than that at the other three altitudes. For instance, the maximum *F*_A_ was 7.39‰ at 30–40 cm soil depth and 1268 m altitude, compared to the values of 3.66‰ to 5.23‰ at the same layer of three lower altitudes. The relative enrichment factor (*F*_R_) was generally higher in the surface soil layers of 0–10 cm (1.49‰ to 4.43‰) and 10–20 cm (0.93‰ to 1.76‰) than in the deeper layers (Fig. 5).

### Relationships of Soil δ^13^C with SOC Concentration and Chemical Composition

**Figure 6:**
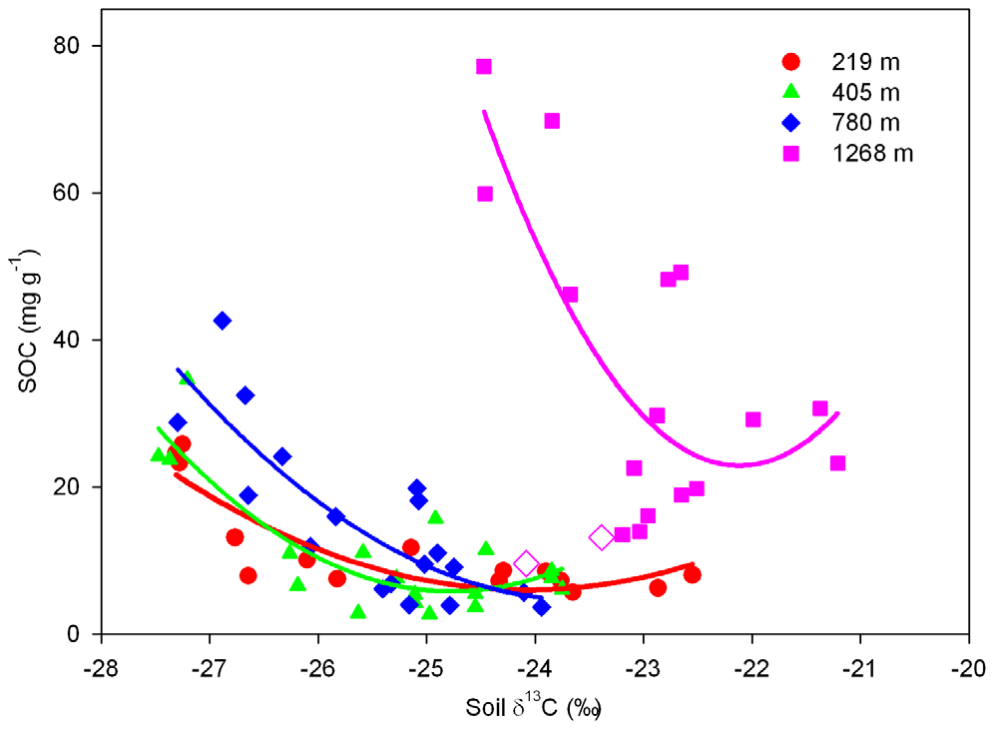
Relationships between SOC concentration (mg g^−1^) and soil δ^13^C by soil depth for four altitudes in the Lushan Mountain. The fitted models are: *y = *870.58+71.82*x* +1.49*x* ^2^, *r* ^2^ = 0.76, and *p* = 0.0002, for the 219 m site; *y* = 1881.58+149.87*x* +3.03*x* ^2^, *r* ^2^ = 0.72, and *p*<0.0001, for the 405 m site; *y* = 1250.35+105.77*x* +2.25*x* ^2^, *r* ^2^ = 0.68, and *p* = 0.0002, for the 780 m site; and *y* = 4265.44+383.58*x* +8.67*x* ^2^, *r* ^2^ = 0.57, and *p* = 0.0041, for the 1268 m site.

**Figure 7:**
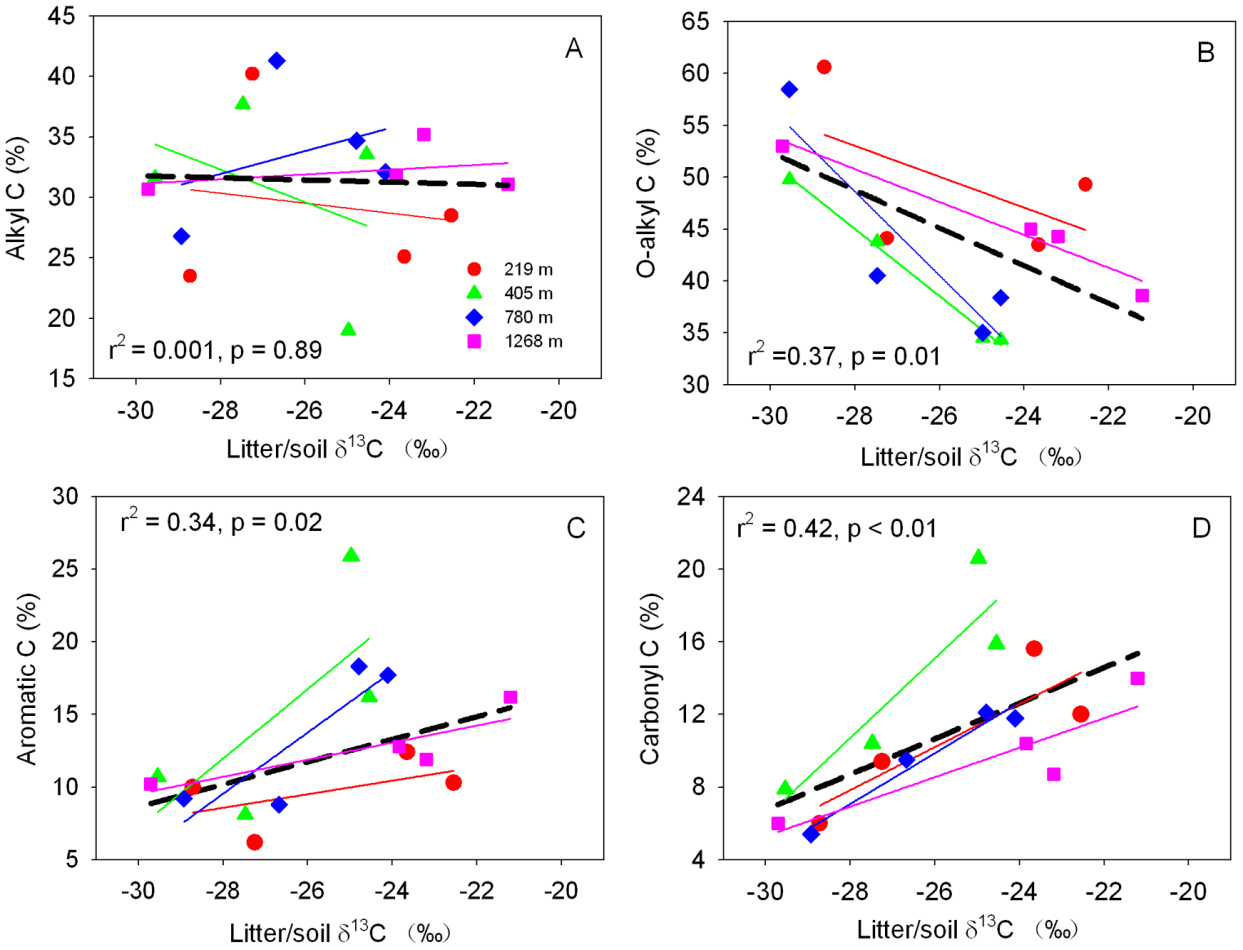
Relationships between soil δ^13^C and Alkyl C (A), O-alkyl C (B), Aromatic C (C), and Carbonyl C (D) for the studied stands at the four latitudes in the Lushan Mountain. Dashed lines represent the general regression lines with all data, with significant level of p<0.05, except for Alkyl C.

Soil δ^13^C increased (Fig. S1) with SOC concentration at each altitude, following a strong negative relationship (*p*<0.01) (Fig. 6). The relationship at the three sites of lower altitudes (219 m, 405 m and 780 m, all covered by evergreen forests) was different from that at the site of highest altitude (1268 m, covered by deciduous forests). Soil δ^13^C was negatively correlated with O-alkyl C, but positively with aromatic C and carboxyl C (Fig. 7).

### Relationships between Soil δ^13^C and the Climatic Factors

**Figure 8:**
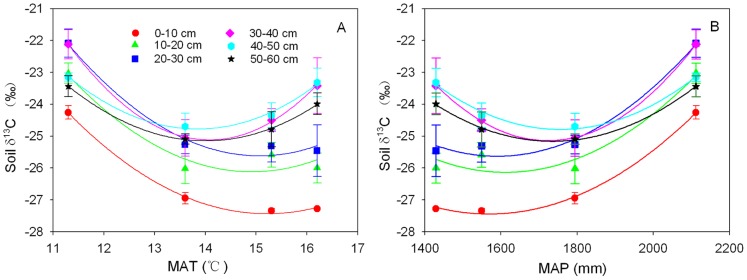
Variations of soil δ^13^C with the mean annual temperature (MAT) (A) and mean annual precipitation (MAP) (B) by soil depth in the Lushan Mountain. The fitted model is y = y_0_+a*x*+b*x* ^2^. The regression lines wtih MAT have *r* ^2^ = 0.998 and *p* = 0.0428 for 0–10 cm layer, and *r* ^2^ = 0.999 and *p* = 0.0289 for 30–40 cm layer (A), and those with MAP have *r* ^2^ = 0.998 and *p* = 0.0445 for 0–10 cm layerand *r* ^2^ = 0.999 and *p* = 0.0175 for 30–40 cm layer (B).

In Lushan Mountain, temperature decreases with, whereas precipitation increases with the increase of elevation. The soil δ^13^C decreased with MAT and increased with MAP in the three upper layers (0–10 cm, 10–20 cm, and 20–30 cm), whereas no clear trend existed for deeper layers (Fig. 8).

## Discussion

### Decoupled Patterns of Variations in Plant and Soil δ^13^C along Elevation

In terrestrial ecosystems, plant functional types strongly affect site-level soil δ^13^C through litter inputs [5], [30], [31]. For instance, Peri et al. (2012) reported that the soil δ^13^C in *Nothofagus* forests was significantly associated with foliar δ^13^C, both of which decreased with precipitation [28]. In the present study, however, fresh leaf and leaf litter δ^13^C decreased with, whereas soil δ^13^C increased with altitude (Figs. 2 and 3), a pattern that cannot be explained alone with the increasing precipitation by altitude in Lushan Mountain.

Different from some previous studies [33], [34], the tree leaf δ^13^C was significantly lower at the highest altitude, likely due to the special climate regime in Lushan Mountain where precipitation increases with and temperature decreases with altitude (Fig. 8). According to a general notion about plant ^13^C discrimination [35], plants at moist sites tend to have high stomatal conductance (close to maximum), low water use efficiency, and high intercellular CO_2_ concentration. This results in increasing discrimination against ^13^CO_2_ during photosynthesis leading to low δ^13^C values, in comparison with arid sites. In Lushan Mountain, plants experience greater drought stress at lower altitude sites owing to low precipitation and high temperature, resulting in high tissues δ^13^C.

In the present study, soil δ^13^C increased with altitude, consistent with the pattern found in previous studies [7], [34], [36], [37], [38]. For example, Townsend et al. (1995) reported an increase of soil δ^13^C from –26.70 ‰ at 900 m a.s.l. to –25.90 ‰ at 1500 m a.s.l. in the island of Hawaii [32]. Similar results are also reported by Zimmermann et al. (2012) in a tropical forest in Peru where soil δ^13^C values increased with elevation from –27.16‰ at 1700 m a.s.l. to –25.79‰ at 3030 m a.s.l. [38]. The major reason for the altitudinal variation of soil δ^13^C in those studies is probably the influence of plant communities through the deposition of leaf litter, dead root material, and rhizodeposition [32]. In Lushan Mountain, however, increasing precipitation and decreasing temperature with altitude may have predominantly influence over soil δ^13^C.

Relative to the bulk leaf δ^13^C, sugars, starch, cellulose, protein, and organic aids are enriched, whereas lignin and lipids are depleted in δ^13^C [6]. Therefore, organic matter with high concentrations of sugars, starch, and cellulose displays high δ^13^C values. On the other hand, however, sugars, starch, cellulose, and protein, are more easily lost through litter decomposition than lignin [39], [40]. This helps explain the high soil δ^13^C of top soil layers (0–10 cm, 10–20 cm, and 20–30 cm) at the altitude of 1268 m (Fig. 3) where high moisture and low temperature not only reduces forest productivity and litter (organic matter) input to the soil, but also shows down decomposing activities of microbes.This may have led to accumulation of more less-decomposed organic matter in soils and therefore high soil δ^13^C (accumulation of sugars, starch, cellulose, and protein).

### Soil δ^13^C Enrichment with Soil Depth by Altitude

In previous studies, enrichment factors (*F*_A_ in this study) were used to describe the variation in soil δ^13^C enrichment relative to litter. However, the results of this study suggest that the relative enrichment factor (*F*_R_) introduced in the present study better detect the difference of soil ^13^C enrichment by depth than *F*_A_ used by previous studies (Figs. 4 and 5). For instance, *F*_R_ shows a more rapid change from semi-decomposed litter to soil layers (0–10 cm, 10–20 cm) than *F*_A_ (Fig. 5).

At all altitudes, soil δ^13^C was enriched with soil depths from litter to O-layer and to mineral soil layers (Figs. 4 and S1). This finding is consistent with the conclusion by previous studies [1], [2], [11], [39]. In the present study, the absolute enrichment factor at 1268 m a.s.l. (*F*_A_ = 5‰ to 7.5‰) was greater than that at lower altitudes (*F*_A_ = 1.5‰ to 5.5‰). For example, The *F*_A_ of 0–10 cm at 1268 m a.s.l. was about 5‰ nearly twich of that of the same soil depth at all lower altitudes (Fig. S1). This result suggests that the climatic conditions (MAP = 2112 mm, MAT = 11.3°C) at 1268 m a.s.l. support a distinct fractionation compared to lower altitudes, particularly the sites at 219 m a.s.l. with MAP = 1429 and MAT = 16.2. Therefore, the climate regime lower temperature and higher moisture at high altitude strongly influences soil ^13^C enrichment with soil depth [41], [42].

### Implications of Soil δ^13^C in Ascertaining SOC Status

Soil C concentrations are strongly correlated with δ^13^C in forest soil, following a negative relationship as demonstrated by previous studies [1], [31], [43]. In this study, soil δ^13^C increased with soil depth, aromatic C and carbonyl C, but decreased with O-alkyl C (Figs. 3 and 7). The increase of ^13^C and changes of SOC chemical composition with soil depth likely result from humification [44], [45]. Microbial activities influence isotopic fractionation during SOC decomposition through differentiation use of substrate by different microbes [41], [42], [46] and isotopic effects on metabolic synthesis of secondary compounds (e.g., lipids, lignin, cellulose) [4], [47]. However, lipids and lignin are degraded more slowly and tend to be ^13^C depleted, whereas cellulose and carbohydrate degrade more rapidly and tend to be ^13^C enriched [42], [48]. Therefore, it is difficult to establish direct relationships between δ^13^C and SOC chemical compositions with soil depth. The distinct high soil δ^13^C at 1268 m a.s.l. is probably attributed to the accumulation of higher ^13^C-based sugars, starch, cellulose, protein, and organic aids, resulting from slow litter decomposition in the surface soil layers (0–10 cm, 10–20 cm, and 20–30 cm) (Fig. 3). Therefore, the enrichment mechanisms of soil ^13^C along the altitude gradient were different from those by soil depth in Lushan Mountain. The detail mechanisms need to be clarified in future research.

## Concluding Remarks

The patterns of plant and soil δ^13^C variations and their relation to the climate regime along an altitudinal gradient were studied in Lushan Mountain of central subtropical China. The results indicated that tree leaf δ^13^C decreased and soil δ^13^C increased with altitude. The decoupled pattern of plant and soil δ^13^C was due to the climate regime of decreasing temperature and increasing precipitation with altitude in the study area, which result in decreased litter decomposition at high-latitude sites. These results have important implications for understanding C dynamics of subtropical forest ecosystems in the context of global warming.

## Supporting Information

Figure S1 **Variation of δ^13^C with litter/soil depth by stands at altitudes of 219, 405, 780, and 1268 m in Lushan Mountain.**
(DOCX)

Table S1 **Site and stand conditions of studied area.**
(DOCX)
