# Supplementary material for: Climatic Control on Plant and Soil δ13C along an Altitudinal Transect of Lushan Mountain in Subtropical China: Characteristics and Interpretation of Soil Carbon Dynamics
Source: PLoS One. 2014 Jan 23;9(1):e86440. doi: 10.1371/journal.pone.0086440 (PMC3900521; doi:10.1371/journal.pone.0086440)
Supplement: Figure S1 — Variation of δ13C with litter/soil depth by stands at altitudes of 219, 405, 780, and 1268 m in Lushan Mountain. (DOCX) [file pone.0086440.s001.docx]

**Supplemental materials**


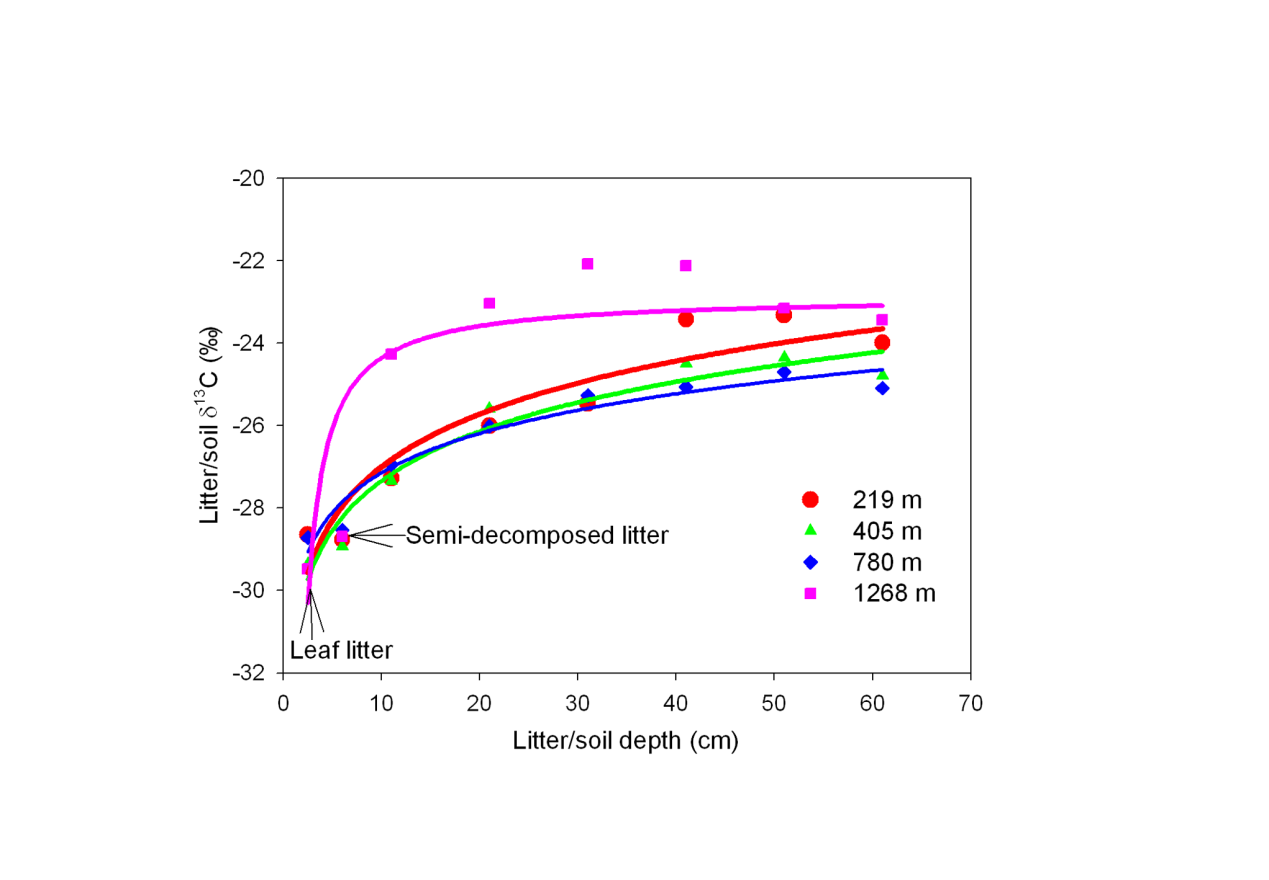


Figure S1. Variations of δ^13^C with litter/soil depth by stands at altitudes of 219, 405, 780, and 1268 m in Lushan Mountain. The fitted models are: y = –31.28 + 1.86 ×ln(abs(x)), r^2^ = 0.89, p = 0.0004, for the 219 m site; y = –31.32 + 1.73 × ln(abs(x)), r^2^ = 0.95, p< 0.0001, for the 405 m site; y = –30.33 + 1.38 × ln(abs(x)), r^2^ = 0.95, p< 0.0001, for the 780 m site; and y = –22.86 × x/(x– 0.61), r^2^ = 0.76, p = 0.0048, for the 1268 m site.

Soil δ^13^C was enriched with increased soil depth, with the peak at 40 - 50 cm layer at altitudes of 219, 405, and 780 m and at 20 - 30 cm layer at 1268 m a.s.l. (Fig. S1). Soil δ^13^C (from layers 0 - 10 cm and 50 - 60 cm) was higher at 1268 m a.s.l. than at other three altitudes. Based on the fitted equations, the soil δ^13^C–depth relationship followed *y* = a + b ln(*x*) at the altitudes of 219, 405, and 780 m (*r*^2^ = 0.89 to 0.95, p < 0.001) and y = a × *x*/(*x* – b) (*r*^2^ = 0.76, p = 0.0048) at the altitude of 1268 m.
