# Supplementary material for: Climatic Control on Plant and Soil δ13C along an Altitudinal Transect of Lushan Mountain in Subtropical China: Characteristics and Interpretation of Soil Carbon Dynamics
Source: PLoS One. 2014 Jan 23;9(1):e86440. doi: 10.1371/journal.pone.0086440 (PMC3900521; doi:10.1371/journal.pone.0086440)
Supplement: Table S1 — Site and stand conditions of studied area. (DOCX) [file pone.0086440.s002.docx]

**Supplemental materials**

Table S1. Site and stand conditions of studied stands.

| Altitude(m) | Plot codes | Slope (%) | Soil types | Dominant tree species | Density (stems ha^-1^) | Height(m) | DBH (cm) | Ground vegetation species |
| --- | --- | --- | --- | --- | --- | --- | --- | --- |
| 219 | I-1 | 35 | Ferric alisols | *C. sclerophylla* | 175 | 12.0 | 35.3 | I-1: *Clerodendrum cyrtophyllum, Pleioblastus amarus,*  *Camellia cuspidate,* *Rubus corchorifolius* |
|  | I-2 | 40 | Ferric alisols | *C. sclerophylla* | 500 | 12.9 | 27.0 | I-2: *C. cuspidate, Eurya japonica, C. cyrtophyllum,*  *Oplismenus undulatifolius* |
|  | I-3 | 25 | Ferric alisols | *C. sclerophylla* | 425 | 16.0 | 30.1 | I-3: *Camellia japonica, Vernicia fordii, E. japonica, Dryopteris labordei, Woodwardia japonica* |
| 405 | II-1 | 45 | Alumi-ferric alisols | *C. sclerophylla,*  *L. glaber* | 875 | 9.8 | 20.3 | II-1: *Litsea cubeba, Radix syzygii, C. cuspidate, Oplismenus undulatifolius* |
|  | II-2 | 40 | Alumi-ferric alisols | *L. glaber* | 575 | 9.3 | 18.4 | II-2: *L. cubeba, R. syzygii, Trema cannabina var. dielsiana, Symplocos caudate, C. cuspidate, Ardisia crenata* |
|  | II-3 | 35 | Alumi-ferric alisols | *L. glaber*, | 1225 | 8.9 | 13.5 | II-3: *L. cubeba, C. cuspidate, C. eyrei, D. labordei, E. japonica* |
| 780 | III-1 | 35 | Haplic alisols | *Pinus massoniana,*  *C. eyrei* | 1125 | 8.1 | 16.4 | III-1: *Camellia oleifera, Rhododendron mariesii, Corylopsis sinensis; Stewartia gemmata* |
|  | III-2 | 45 | Haplic alisols | *C.eyrei* | 1375 | 9.5 | 16.6 | III-2: *C. cuspidate, Ternstroemia gymnanthera, R. mariesii, Photinia serrulata, Acerdavidii Franch* |
|  | III-3 | 35 | Haplic alisols | *C. eyrei* | 625 | 9.4 | 21.7 | III-3: *Rhododendron delavayi, Pterostyrax corymbosus, Lindera reflexa, C. oleifera* |
| 1268 | VI-1 | 30 | Haplic alisols | *L. obtusiloba* | 1250 | 6.3 | 7.0 | VI-1: *Cyclobalanopsis glauca, Pteroceltis tatarinowii, Toxicodendron succedaneum, Hydrangea angustipetala, Weigela japonica var. sinica , Aster ageratoides* |
|  | VI-2 | 45 | Haplic alisols | *L. obtusiloba* | 1200 | 5.0 | 9.0 | VI-2: *C. glauca, P. tatarinowii, T. succedaneum, Cerasus serrulata, Spiraea chinensis, A. ageratoides* |
|  | VI-3 | 45 | Haplic alisols | *T. breviradiata* | 1725 | 7.7 | 8.3 | VI-3: *P. tatarinowii, T. succedaneum, Viburnum fordiae, R.mariesii, A. ageratoides, Astilba chinensis* |

Diameter at breast height (DBH, 1.3 m above the ground) was measured for the trees ≥ 4.8 cm d.b.h. (15 cm girth). The density of each species was calculated as the number of individuals per sampling site. Tree height was estimated visually and checked occasionally using an ultrasonic altimeter.
